# A Fast, Visual, and Instrument-free Platform Involving Rapid DNA Extraction, Chemical Heating, and Recombinase Aided Amplification for On-Site Nucleic Acid Detection

**DOI:** 10.3389/fbioe.2021.764306

**Published:** 2021-11-22

**Authors:** Xiao Fu Wang, Wen Qiang Chen, Jian Li Guo, Cheng Peng, Xiao Yun Chen, Xiao Li Xu, Wei Wei, Lei Yang, Jian Ca, Jun Feng Xu

**Affiliations:** ^1^ State Key Laboratory for Managing Biotic and Chemical Threats to the Quality and Safety of Agro-products, Zhejiang Academy of Agricultural Sciences, Hangzhou, China; ^2^ Key Laboratory of Information Traceability for Agricultural Products, Ministry of Agriculture of China, Hangzhou, China; ^3^ College of Biological and Food Engineering, Fuyang Normal University, Fuyang, China

**Keywords:** rapid DNA extraction, chemical heating, RAA, on-site detection, nucleic acid

## Abstract

The nucleic acid-based technique has been widely utilized in many fields including for on-site detection. However, traditional molecular detection techniques encounter limitations like relying on instruments, time consuming or complex operation, and cannot meet the demands of on-site testing. In this study, a rapid DNA extraction method (RDEM), recombinase aided amplification (RAA), and chemical heating packet (CHP) are integrated and termed as RRC platform for on-site detection of nucleic acid. For demonstration purposes, SHZD32-1 (a new transgenic soybean line from China) was detected using the novel platform to demonstrate its feasibility and capability for on-site detection. Using the RDEM, high-quality DNA appropriate for molecular detection was quickly extracted in 3–5 min. The heat energy generated by CHP was met the temperature requirements of RAA. Using the RRC platform, the whole detection process can be accomplished within only 30 min, and the results can be visually detected with glasses under blue light. No special or expensive instrument was needed for the detection process. This study provides a novel approach for on-site detection of nucleic acids besides providing valuable insight on related future research.

## Introduction

At present, numerous nucleic acid-based analytical techniques were developed. It has been extensively applied in many fields including disease detection, food safety, and environmental monitoring (Van Nguyen et al., 2019; Zhang et al., 2020). However, there are some limitations in on-site detection or diagnosis using nucleic acid-based methods.

The nucleic acid-based method generally comprises three steps including nucleic acid extraction, amplification, as well as product detection. Nucleic acid extraction is the most critical step in the on-site detection of nucleic acid. To date, two methods have been applied in the extraction of nucleic acid. The most commonly used method is the DNA extraction method, which entails the traditional reagent extraction and commercial kit extraction techniques. These DNA extraction techniques are time-consuming (1–2 h), and require auxiliary equipment like water bath and centrifuge (Lodhi et al., 1994; Zhang et al., 2013). As such, this method is unsuitable for on-site detection which should be fast and does not rely on large instruments. To solve this problem, another method referred to as DNA extraction-free method was utilized. The samples were treated with cell lysate and then the supernatant of the treatment solution worked as the templates in the DNA amplification step. Although this treatment is rapid and convenient, it is unstable for plant tissues since they contain more secondary metabolites, which would affect the efficacy of the DNA amplification process. As such, it is important to carry out further research on the methods of nucleic acid extraction to facilitate on-site detection of the nucleic acid.

With regard to amplification, polymerase chain reaction (PCR) is the most popular method. It is consider as the universal standard for molecular detection. However, the thermal cycling steps in large and expensive equipment required by PCR hinder its application in on-site detection. Isothermal nucleic acid amplification (INAA) has emerged as a potential alternative for PCR. In INAA, rapid amplification is achieved at a constant temperature and does not require large-scale equipment. Currently, a large number of INAA-related studies have been published, such as recombinase aided amplification (RAA) (Piepenburg et al., 2006), cross-priming amplification (CPA) (Xu, et al., 2012), loop-mediated isothermal amplification (LAMP) (Notomi, et al., 2000), and rolling circle amplification (RCA) (Lizardi, et al., 1998). Among these INAA methods, RAA is an extraordinary isothermal system that simulates *in-vivo* DNA replication (Piepenburg, et al., 2006). Initial denaturation is not required for the template DNA, so it can be carried out under a relatively low temperature (about 39°C). Exponential amplification can be accomplished within a short time. RAA technology is increasingly used in many fields such as detection of DNA, RNA virus, and fungal pathogens etc. (Lobato and O'Sullivan, 2018), and has demonstrated high applicability for on-site detection (Chen et al., 2020; Zhang, et al., 2021).

The last step entails the production detection. Agarose gel electrophoresis is the traditional method used for the production detection. New production detection methods like DNA intercalating dye, fluorescence probe, lateral flow dipstick, and nanoparticle have been discovered. However, these methods exhibit some limitations in on-site detection, including: the DNA dye would affect the efficiency of the amplification, fluorescence probe requires a special instrument to monitor the fluorescence signal, and the preparation either lateral flow dipstick or nanoparticle is complicated and expensive. Regarding the on-site detection, to meet the requirement of applications in field detection and poor-resource areas, the detection process should be simple, rapid and instrument-free.

**SCHEME 1:**
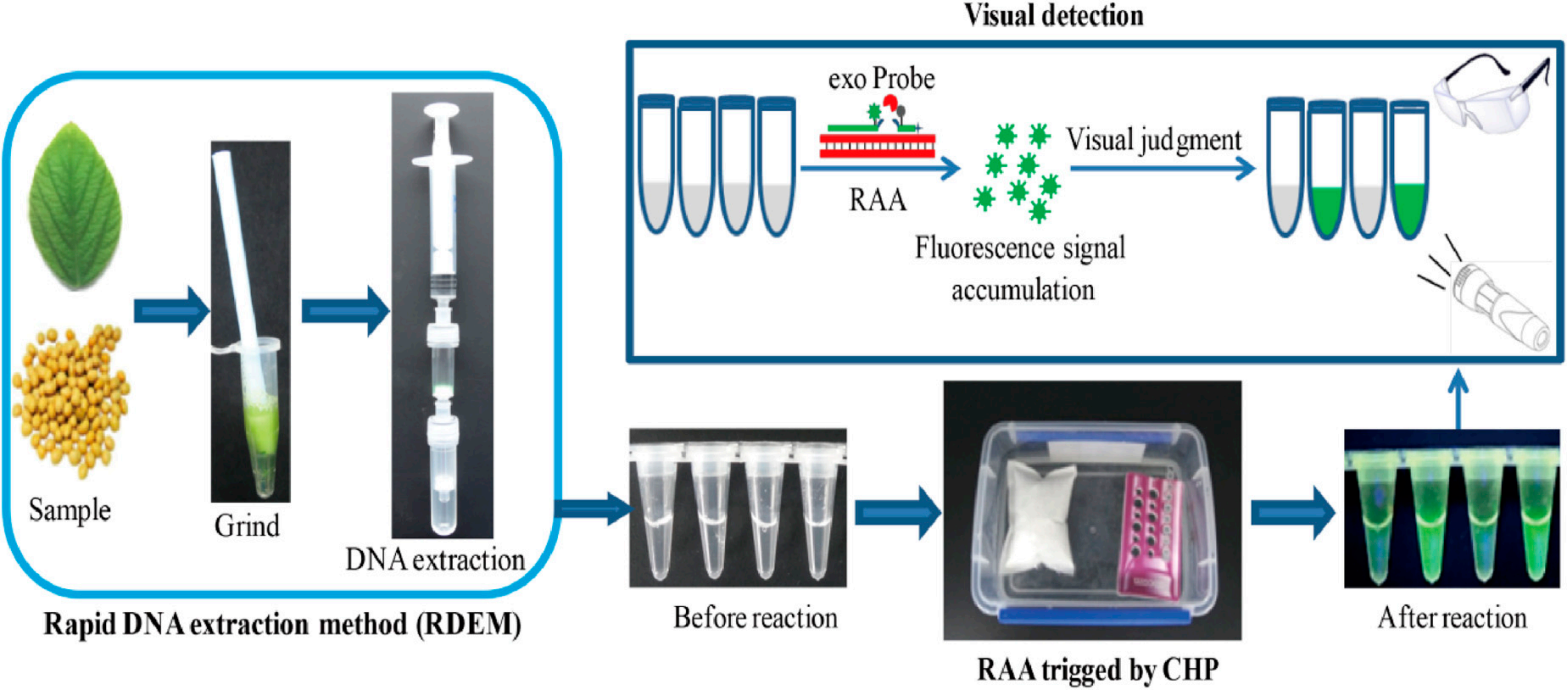
Schematic diagram of the RRC platform.

In this study, putting into account the challenges encountered in the three steps of nucleic acid-based detection method, we developed a simple, fast, visual, as well as instrument-free platform for on-site detection of nucleic acid. We combined the rapid DNA extraction method (RDEM) with the recombinase aided amplification (RAA), trigged by the chemical heating packet (CHP), as a platform for on-site detection of the nucleic acid. This detection platform was regarded as RRC (RDEM and RAA and CHP), and the major steps of the platform are shown in Scheme 1. The on-site detection capability of RRC platform was evaluated via the detection of a genetically modified (GM) soybean SHZD32-1, which was the first transgenic soybean line to obtain a safety certificate in China.

## Materials and Methods

### Materials

GM soya SHZD32-1 and non-GM soya were obtained from the Shanghai Jiao Tong University. Other transgenic crops, including GM soya: A2704-12, A5547-127, 356,043, GTS40-3-2, and FG72; GM maize: NK603, BT176, and T25; GM cotton: LLcotton25, MON15985, and GHB119; and GM rapeseed: MS1×RF1, MS1×RF2, and OXY235, were provided by the Shenzhen Excellent Biotech Co., Ltd. (Shenzhen, China). The recombinase aided amplification (RAA) kit was purchased from Weifang Amp-future Biotech Co., Ltd. (Weifang, China). The aperture and thickness of the filter membrane were 10 μm and 1 mm, respectively. The aperture of the silica membrane was 1 μm and its thickness was 0.3 mm. The pore size of column was 7.25 mm. All these materials were bought from Lifefeng Biotechnologies (Hangzhou) Ltd.

### DNA Extraction by the Rapid Extraction Method

**FIGURE 1:**
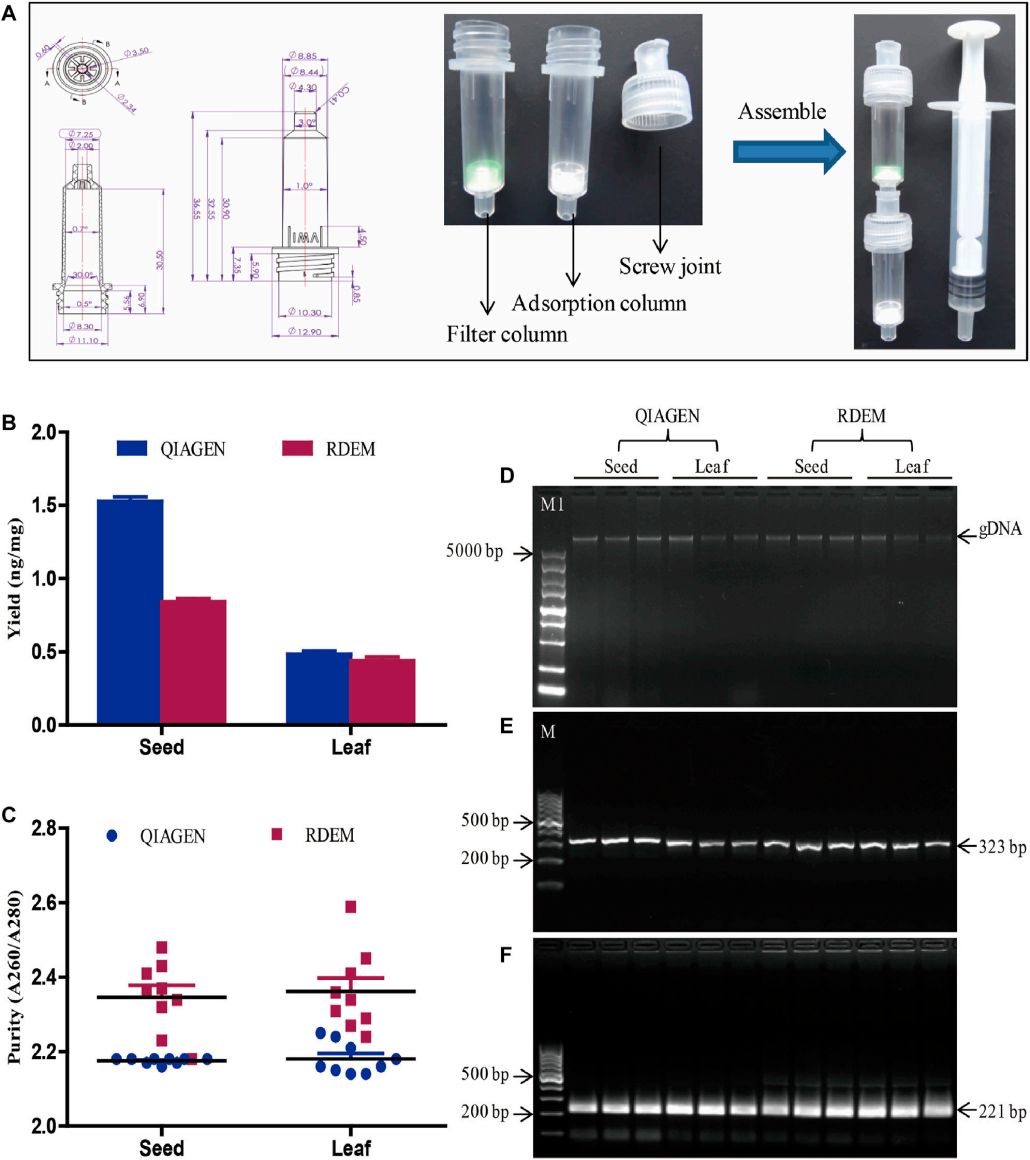
DNA extraction and evaluation. **(A)** Diagram of the RDEM. **(B)** Comparison of DNA yields of the QIAGEN kit and RDEM. **(C)** Comparison of DNA purity of the QIAGEN kit and RDEM. **(D)** Agarose gel electrophoresis of the extracted genomic DNA from the seed and leaf using the QIAGEN kit and RDEM. **(E)** Conventional PCR amplification of the 18S rRNA gene from all the extracts. **(F)** Basic RPA amplification of 18S rRNA gene from all the extracts. M1: DL5000 DNA marker, M: DL1000 DNA marker.

The rapid extraction method was based on the fact that DNA can be adsorbed and eluted by silicon membrane under various salt concentrations. Air pressure from a syringe was used to accelerate the filtration. The rapid extraction method comprised three devices, including a filter column, adsorption column, and syringe (Figure 1A). Regarding the on-site test of leaf samples, approximately 100 mg of fresh soya leaves and 800 μL of DNA extraction buffer (5 M guanidine thiocyanate, 50 mM Tris, 20 mM EDTA, 21.3 mM Triton X-100, pH 6.4) were put into a 1.5 ml Eppendorf tube. A disposable plastic pestle was used to homogenize the mixture by stirring it for 1 min. Later the homogenized mixture was transferred to a filter column. The filter column was joined to the syringe and the adsorption column using screw joints. The syringe plunger was pressed to filter the mixture through the filter membrane and into the adsorption column. The adsorption column was put into a new 2 ml tube, and 400 μL a wash buffer A (5 M guanidine thiocyanate, 50 mM Tris, pH 6.4) was passed through the adsorption column, so as to wash the silica membrane. Then 200 μL of a wash buffer B (10 mM Tris, 100 mM NaCl, pH 8.0) was added to the adsorption column to rewash it. The adsorption column was placed into a new 2 ml tube, and 100 μL TE buffer was added to the adsorption column by using a syringe to elute the DNA on the silica membrane. With regard to the soya seeds samples, the seeds were smashed to powder by a hammer, and seed powder (approx. 100 mg) was transferred to a 2 ml tube. Then the DNA extraction from seed powder was performed as described above for the leaf sample. Simultaneously, the DNeasy Plant Mini Kit (Qiagen, Hilden, and Germany) was used in extraction of similar samples to compare with the performance of the rapid DNA extraction method.

### DNA Amplification Reactions and Product Detection

The RAA reactions were conducted based on to the manufacturer’s guidelines (Exo kit/basic kit, Amp-future Biotech, Weifang, China). The total volume of the RAA system was 50 µL. It consisted of 29.4 µL buffer, 14 mM magnesium acetate, 1 µL DNA template, one lyophilized enzyme pellet, 400 nM primers with or without 120 nM probe, and nuclease-free water up to 50 µL. The schematic of RAA and it’s exo probe was provided in Supplementary Figure S1. The optimal combination of the primers with the probe was selected (Supplementary Figure S2). The conventional PCR and RT-PCR used in this study were based on previous studies (Wang, et al., 2017, 2020). All the primers and probes were produced by Sangon Biotech Co., Ltd. (Shanghai, China). Detailed information on all the primers and probes is as described in Supplementary Table S1.

For product detection, the results of basic RAA and conventional PCR were analyzed by agarose gel electrophoresis. The real-time fluorescence signal of RAA with exo-probe and RT-PCR were monitored by a Bio-Rad CFX96 RT-PCR Detection System (Bio-Rad Company, Pleasanton, CA, United States). The visual green fluorescence result was obtained with glasses under a hand-held lamp (LUYOR-3415RG).

### Preparation of the Chemical Heating Packet

The principle of the chemical heating packet was that the chemical reagents in the packet reacted with water to produce heat energy. The total amount of chemicals in the packet was 15 g. It comprised 5.4 g Aluminum powder, 3.75 g CaO, 3.3 g CaCO_3_, 1.8 g CaOH, 0.6 g NaCO_3_, and 0.15 g NaOH. All the chemicals were wrapped in non-woven fabric, whose size was 8 cm × 10 cm. The packet was then sealed with oriented polypropylene (OPP) film. For the on-site detection, the packet was put in a vessel (length 19 cm×width 12 cm×depth 7 cm) after removing the OPP film. Then 350 ml water was put in the vessel to react with the chemical reagents in the packet so as to generate heat.

## Results and DISCUSSION

### Evaluation of the Applicability of the Rapid DNA Extraction Method

In most DNA extraction methods, centrifugation is a vital, indispensable step. The requirement for centrifugal equipment hindered the applicability of these DNA extraction methods for the on-site sample analysis. To meet the requirement of on-site analysis, we came up with a rapid DNA extraction method (RDEM) that did not require centrifugation. The RDEM consisted of a threaded column and a screw joint. The threaded column was connected to the screw joint by the screw thread. The detailed size dimensions of the threaded column and the screw joint are as indicated in Figure 1A. The filter membrane was placed at the bottom of the threaded column, to form a filter column, to filter the plant residues after grinding. The adsorption columns were fitted with a silica membrane to adsorb DNA. The filter column, adsorption column, and syringe were connected with screw joints (Figure 1A).

To determine the feasibility of the RDEM, soybean leaves and seeds were used in DNA extraction. To compare the findings, similar extraction was also performed using a commercial DNA extraction kit (DNeasy Plant Mini Kit, Qiagen). The quantity and purity of the DNA extracted using the two independent methods are indicated in Figures 1B,C. The average DNA yields of the seed and leaf were 1.52 and 0.48 ng/mg, respectively, based on the QIAGEN kit. On the other hand, the mean DNA yields of the seed and leaf were 0.84 and 0.44 ng/mg, respectively, according to our RDEM (Figure 1B). The OD A260/280 ratios of the DNA extracted from the seed and leaf were 2.17 and 2.34 as indicated by the QIAGEN kit, whereas they were 2.34 and 2.36, respectively, from the RDEM analysis (Figure 1C). Both the yield and purity of the DNA extracted using RDEM were lower than those extracted using the QIAGEN kit. The integrity of the DNA extracted by the two techniques was similar, as shown in agarose gel electrophoresis (Figure 1D). Besides, to ascertain if the DNA extracted by RDEM was appropriate for DNA amplification, the DNA extracted using the two methods was used as a template to amplify the endogenous plant gene (18S rRNA) (Wang, et al., 2020), via the conventional PCR and basic RAA. The expected size of the 18S rRNA gene was amplified and observed for all the samples in the PCR and RAA assays (Figures 1E,F). These findings suggested that, although the yield and purity of the DNA extracted using RDEM were not as high as those of the QIAGEN kit, they were sufficient for the subsequent molecular tests like PCR and RAA.

### Chemical Heating Triggers RAA Reaction

**FIGURE 2:**
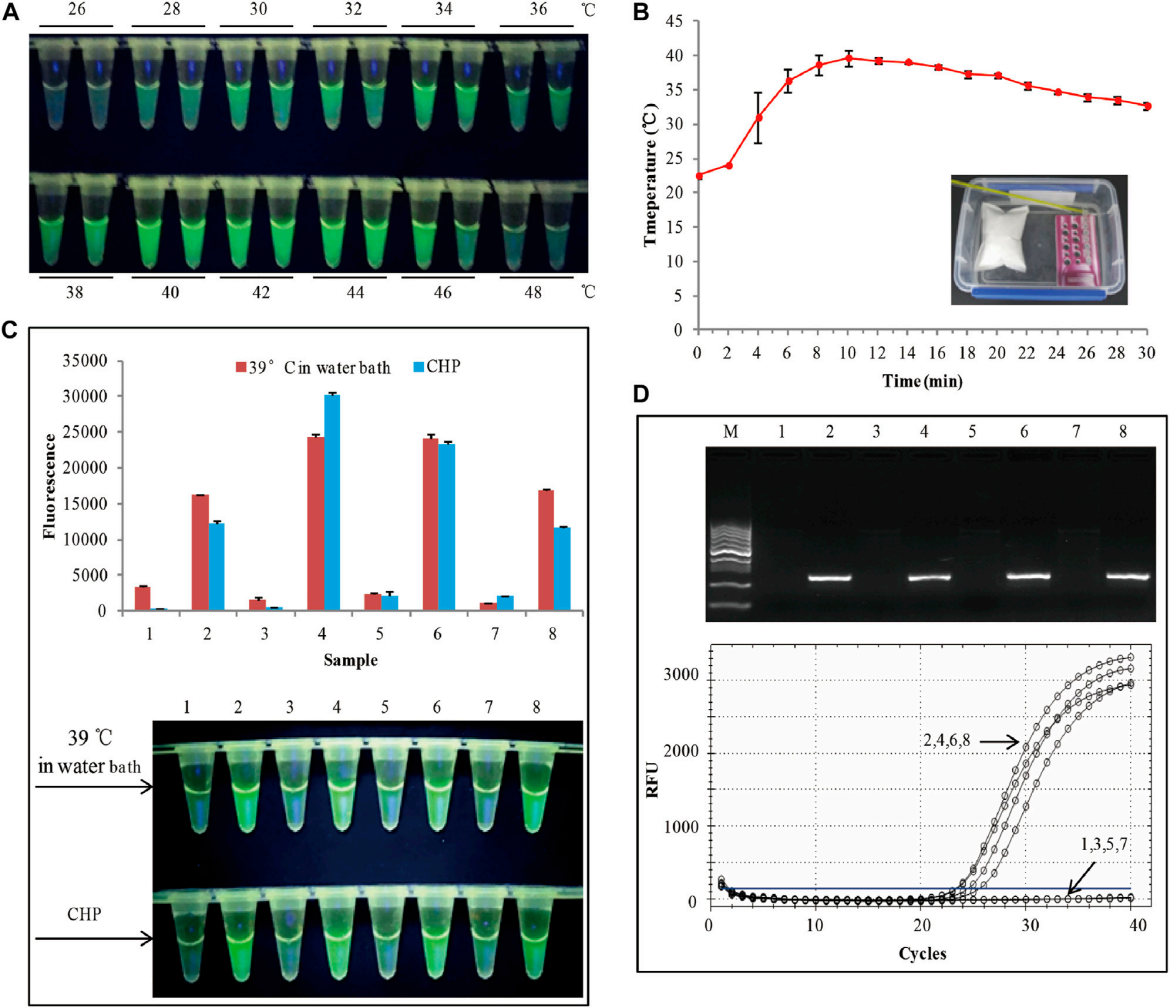
**(A)** Assessment of the RAA temperature range. **(B)** Water heated by the chemical heating packet for 30 min. **(C)** The RAA was triggered by a water bath at 39°C. The **top panel** shows the end-point fluorescence values of the two RAA assays. The **bottom panel** shows the visual fluorescence outcomes of the two RAA assays. **(D)**The detection results of conventional PCR and RT-PCR. 1,3,5,7: non-GM soya, 2,4,6,8: SHZD32-1, M: DL1000 DNA marker.

According to previously reported (Boyle, et al., 2013; Lillis, et al., 2014), RAA has a wide range of reaction temperatures. In this study, the wide range of temperatures whether suitable for visual fluorescence detection was evaluated. RAA conducted by amplifying 40 copies of genomic DNA from SHZD32-1 at various temperatures for 20 min (Figure 2A). The visual fluorescence finding of this assay, from 28 to 46°C, indicated a green fluorescence with varying intensities from each reaction. The strongest fluorescence was observed between 34 and 44°C. At 26 and 48°C, no green fluorescence was observed in the RAA reaction (Figure 2A). The probable reasons behind these observations were: at 26°C, the temperature was too low to activate the enzymes involved in the RAA reaction. On the other hand, at 48°C, the temperature was too high and denatured the enzymes thus hindering the enzymatic activity in the RAA reaction. These findings confirmed that RAA has a wide range of reaction temperatures and also suitable for visual fluorescence detection.

Based on the above findings, we decided to apply chemical heating to stimulate the RAA reaction. To meet the temperature range for the RAA reaction, we adjusted the amount of each chemical composition in the chemical heating packet (CHP). With an ambient temperature of 24°C, at 2 min, the water temperature was 24°C, and at 4 min, it rose to 31° (Figure 2B). From 4 to 24 min, the mean water temperature was 37°C. Besides, the heating temperature by the CHP was also based on the ambient temperatures of 4°C and 34°C, respectively (Supplementary Figure S3). The results indicated that from 4 to 24 min, the mean water temperature was >36°C. This met the RAA temperature requirements. We used the chemical heating packet (for 4–24 min) to activate the RAA reaction, and 4 SHZD32-1 and 4 non-GM soybeans were the testing samples. Similar RAA reaction was performed at 39°C in a water bath for 20 min. The visual fluorescence results of the two RAA reactions were similar. In both reactions, a bright green fluorescence was observed in the tube with SHZD32-1, whereas no fluorescence was observed in the tube with non-GM soybean (Figure 2C and Supplementary Figure S3). Simultaneously, the endpoint fluorescence values (using Cytation 5, BioTek, Instruments, Inc., Winooski, VT, United States) of the two distinct RAA reactions were compared, and it was realized they had similar fluorescence values (Figure 2C). At the same time, conventional PCR and RT-PCR were used to detect the same samples, and the detection outcomes were consistent with the result of the RAA assay (Figure 2D). These results suggested that the chemical heating packet that we designed met the temperature requirements of RAA.

Besides, the specificity and sensitivity of the RAA for SHZD32-1 was evaluated using the RRC platform (Supplementary Figure S4). The results demonstrated that the designed RRC platform was highly specific and sensitive. It was hence appropriate for on-site detection of SHZD32-1.

### Real Sample On-Site Detection Using RRC Platform

**FIGURE 3:**
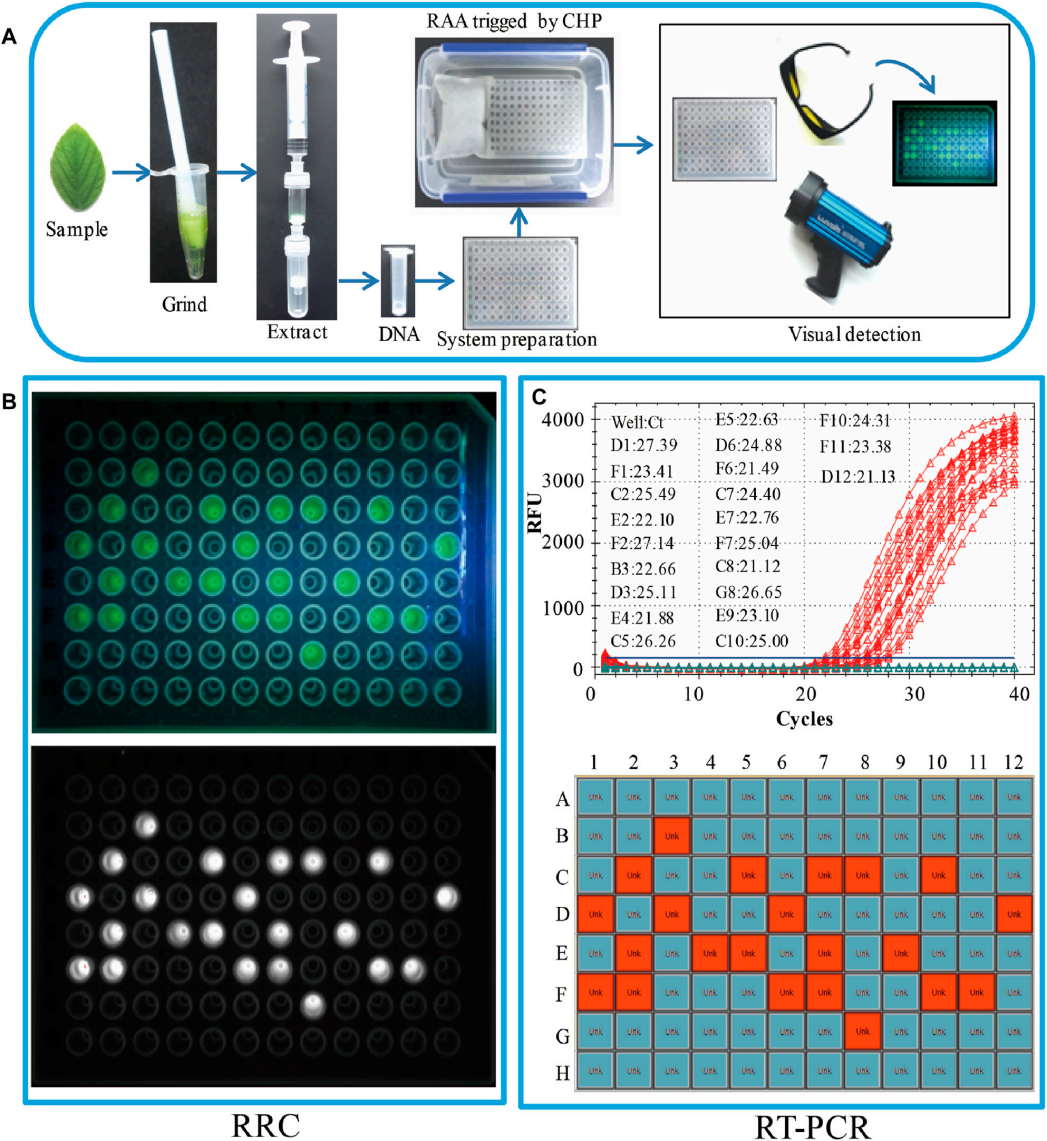
Real sample on-site detection by the RRC platform. **(A)** The workflow of the RRC platform for on-site detection. **(B)** The visual fluorescence result of the RRC platform. The visual detection result under blue light **(top panel)**. The visual detection result of a fluorescence imaging system **(bottom panel)** . **(C)** The detection results of RT-PCR. The amplification curve and Ct value **(top panel)**. The position of positive sample in the 96-well plate **(bottom panel)**.

To evaluate the applicability of the RRC platform in on-site detection of real samples, 96 leaves from 96 soybeans were used as the real samples. A brief workflow is demonstrated in Figure 3A. All DNA was extracted by RDEM, and used as the template for both the RT-PCR and RAA. The visual fluorescence result of the RRC platform indicated that, 22 SHZD32-1 were detected from the 96 samples. The bright green fluorescence presented in the tubes with SHZD32-1. It was therefore easy to distinguish from other tubes without SHZD32-1 (Figure 3B). Besides, the 96-well plate of the RRC platform was analyzed using a fluorescence imaging system (ChemiDoc Imaging System, Bio-Rad, United States). A white fluorescence was observed in these tubes (Figure 3B). This outcome implied that the visualized result of the handheld fluorescent lamp was comparable to the observation result by a large professional instrument. In addition, the on-site detection findings of the RRC platform and RT-PCR were consistent. The same 22 samples were realized to be SHZD32-1 from the 96 samples based on the RT-PCR assay, and the Ct values of the 22 samples were given along with the amplification curve (Figure 3C). These findings suggested that the RRC platform is highly accurate for on-site detection of target nucleic acid.

## Conclusion

We developed a new platform (RRC) to combine a rapid DNA extraction method (RDEM) with RAA trigged by a chemical heating packet (CHP) for on-site detection. The whole detection process was completed within 30 min. Besides, the detection result could be directly observed with glasses, and no need open tubes that eliminated the carryover contamination. The RRC platform demonstrated a high specificity as well as sensitivity. In addition, the entire detection process of this platform was rapid and independent of any equipment. As such, the proposed platform suggests a high potential applicability in many fields, and the development of this platform provides valuable insight on the establishment of a new on-site nucleic acid detection technique.

## Data Availability

The original contributions presented in the study are included in the article/Supplementary Material, further inquiries can be directed to the corresponding authors.
