# Supplementary material for: A Fast, Visual, and Instrument-free Platform Involving Rapid DNA Extraction, Chemical Heating, and Recombinase Aided Amplification for On-Site Nucleic Acid Detection
Source: Front Bioeng Biotechnol. 2021 Nov 22;9:764306. doi: 10.3389/fbioe.2021.764306 (PMC8645684; doi:10.3389/fbioe.2021.764306)
Supplement: Supplementary file 1 [file DataSheet1.docx]

**Supplementary information for**

**A fast, visual, and instrument-free platform involving rapid DNA extraction, chemical heating, and recombinase aided amplification for on-site nucleic acid detection**

Xiao Fu Wang^1,2^, Wen Qiang Chen^1,3^, Jian Li Guo^1^, Cheng Peng^1,2^, Xiao Yun Chen^1,2^, Xiao Li Xu^1,2^, Wei Wei^1^, Lei Yang^1^, Jian Ca^3^* and Jun Feng Xu^1, 2^*

^1^ State Key Laboratory for Managing Biotic and Chemical Threats to the Quality and Safety of Agro-products, Zhejiang Academy of Agricultural Sciences, Hangzhou, China

^2^ Key Laboratory of Information Traceability for Agricultural Products, Ministry of Agriculture of China, Hangzhou, China

^3^ College of biological and food engineering, Fuyang Normal University, Fuyang, China

^*^Corresponding authors:

E-mail: [fycaijian@163.com](mailto:fycaijian@163.com) (Jian Cai), njjfxu@163.com (Junfeng Xu).

**SI-1** ***The principle of RAA and exo-probe***

RAA is a novel isothermal system, in this system, UvsX and primer combine as a complex to scan template DNA and strand exchange is facilitate at cognate sites. The resulting structures are stabilized by GP 32, a single-stranded DNA binding protein. Then UvsX disassembly leaves the 3’-end of the primer, and the primer was extend along another single-stranded DNA by *Bsu* Polymerase. Through circulation of this process, exponential amplification is accomplished. In this study, in order to facilitate the fluorescence observation and improve the specificity and sensitivity of the assay, exo-probe was introduced into this system. In this probe, a nucleotide was replaced by tetrahydrofuran (THF), which was flanked by a fluorophore and a quencher. The THF residue was cleaved by exonuclease III only when the probe was bound to its target. With the cutting step, the fluorophore and quencher were separated, thus generating a fluorescence signal.

**
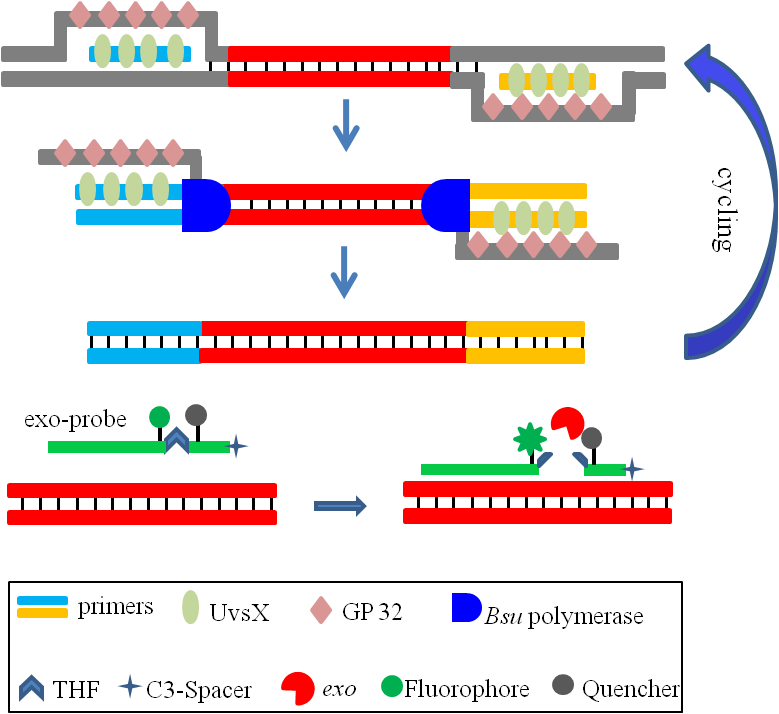
**

**Fig. S1.** Schematic of RAA amplification and exo-probe.

**SI-2 *Primers and probes in this study***

To select optimal primers and probe for specific detection of the SHZD32-1 event with RAA, four forward primers and four reverse primers at each side of the integration junction were designed, along with a exo-probe spanned the junction between the soya genomic sequence and the exogenous element. The locations of the primers and probe at flanking sequences are shown in Fig.S2A. Conventional PCR primers and RAA primers for the 18S rRNA gene were synthesized according to a previous report (Wang et al., 2020). Primers and probe for specific detection of the SHZD32-1 using conventional PCR and RT-PCR were synthesized according to previous study (Wang et al., 2017). All primers and probes were synthesized by Sangon Biotech Co., Ltd. (Shanghai, China). Detailed information on all primers and probes is provided in Table S1.

Table S1 Primers and probes used in this study

| Purpose | Name | Sequence (5' - 3') | Amplicon/bp | Reference |
| --- | --- | --- | --- | --- |
| RAA for SHZD32-1 | SHZD32-1-F1 | CAAAGTCACTCATTATTGGAACCCTACACATC |  | This study |
|  | SHZD32-1-F2 | CCTTCGCTGTTGCAACTCATTGCACAAAGACC |  |  |
|  | SHZD32-1-F3 | CTACACATCCCCTTCCCCCTTCGCTGTTGC |  |  |
|  | SHZD32-1-F4 | CGTCTATTTCATTAACTTTAGGATGTTGCTAA |  |  |
|  | SHZD32-1-R1 | CTTAGTATGTATTTGTATTTGTAAAATACTTC |  |  |
|  | SHZD32-1-R2 | CTTCTATCAATAAAATTTCTAATTCCTAAAAC |  |  |
|  | SHZD32-1-R3 | TAAAATTTCTAATTCCTAAAACCAAAATCCAG |  |  |
|  | SHZD32-1-R4 | AGGGTTCCTATAGGGTTTCGCTCATGTGTTGA |  |  |
|  | SHZD32-1-P | tgttgctaagcacatgcattttaacgaa/FAM-dT/t/THF/at/BHQ1-dT/cgggggatctgg-C3 Spacer |  |  |
| RAA for 18S | RAA-18S-F | TCCTATTGTGTTGGCCTTCGGGATCGGAGTA |  | Wang et al., 2020 |
|  | RAA-18S-R | GATCCCTGGTCGGCATCGTTTATGGTTGAGA | 221 |  |
| Conventional PCR for 18S | PCR-18S-F | CGGGATCGGAGTAATGATTAA |  |  |
|  | PCR-18S-R | CCTGGTGGTGCCCTTCCGTCA | 323 |  |
| Conventional PCR for SHZD32-1 | SHZD32-1-CF | GAGCAGCTTGAGCTTGGA |  | Wang et al., 2017 |
|  | SHZD32-1-CR | CGAATTTCACCAAAACACTAA | 234 |  |
| RT-PCR for SHZD32-1 | SHZD32-1-qF | TCGTTTCCCGCCATAAGG |  |  |
|  | SHZD32-1-qR | CATCAACCAAGAGCAACAGCAT | 120 |  |
|  | SHZD32-1-qP | FAM-TCCGACCACCACGAGACCGTAGTACA-BHQ1 |  |  |

**SI-3 *Selection of the optimal primer pair for RAA***

The amplification efficiency of RAA depends on the primers used in the reaction. However, at present, there is no uniform principles and software for primer design for RAA. In this study, the optimal primer pair and probe for RAA was selected by testing various primer combinations with the probe. First, the forward primer F1 with each reverse primer (R1-R4) was tested in RAA. The primer combinations of F1/R1 and F1/R4 had almost no amplification and the fluorescence signal is very weak. Both the primer combinations of F1/R2 and F1/R3 were amplified, and the amplification efficiency of the F1/R2 primer combination was higher than the F1/R3 primer combination (Fig.S2B). Then, the reverse primer R2 combining with all forward primer F1, F2, F3, and F4 were also tested in RAA. All primer combinations were amplified and fluorescence signal was present for each RAA assay. The final fluorescence intensity of primer combination R2/F1, R2/F2, R2/F3, and R2/F4 was about 7500, 6000, 9000, and 3000, respectively. The time for each combination to reach the fluorescence threshold was about 5 min. From 5 min to 15 min, the R2/F1 combination had the highest amplification efficiency (Fig.S2C). Basing on these results, the F1/R2 was selected as the optimal primer combination, and then used it in subsequent RAA reactions for specific detection of SHZD32-1.

**
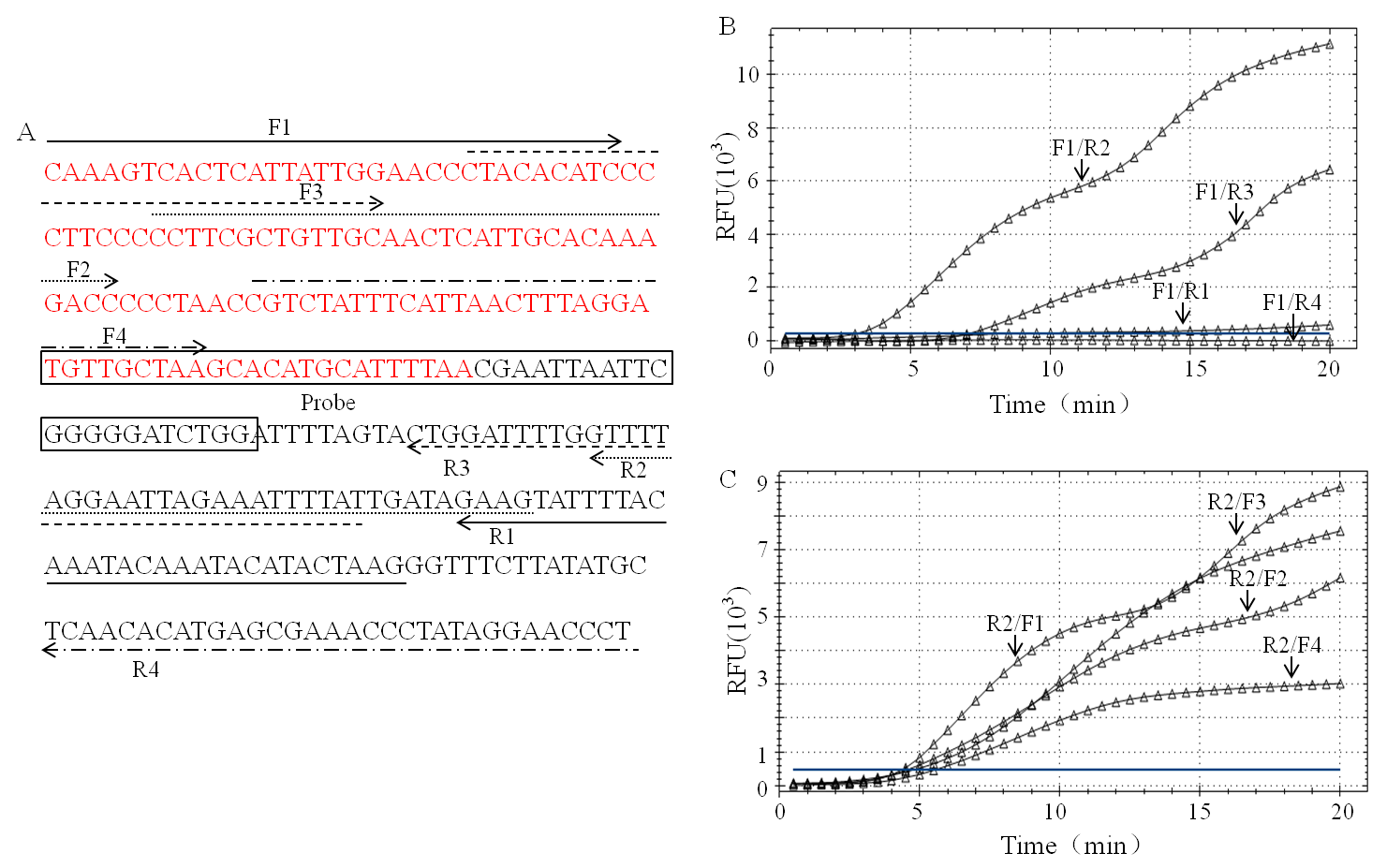
Fig. S2.** Scheme of RAA primers and probe, and the results of primer screening. The locations of the primers and probe on the flanking sequence of SHZD32-1. The soybean genome sequence is red, and the exogenous sequence is black (A). The amplification results of forward primer F1 and all reverse primers (B). The amplification results of reverse primer R2 and all forward primers (C).

**SI-4 Using CHP under different ambient temperature**

The temperature of water heating by the CHP measured under the ambient temperature of 4 °C and 34 °C, respectively, and the RAA reaction was carried out in corresponding water.


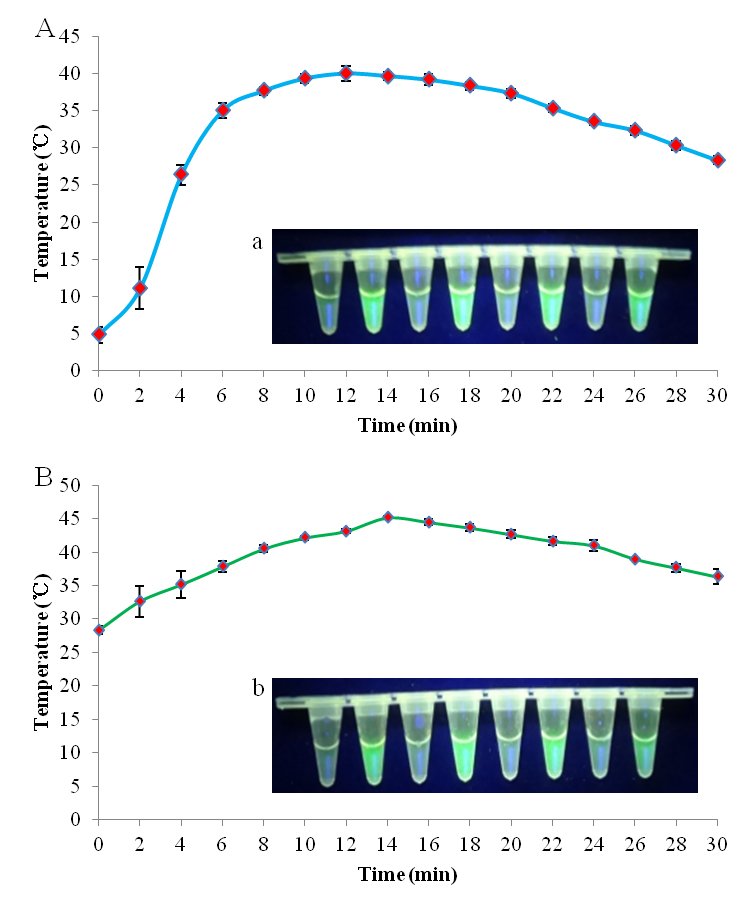


**Fig.S3.** The temperature of the water heated by the chemical heating packet in 30 min under ambient temperature of 4 °C (A), RAA reaction was carried out in the water (a). The temperature of the water heated by the chemical heating packet in 30 min under ambient temperature of 34 °C (B), RAA reaction was carried out in the water (b).

**SI-5 *Specificity and sensitivity of the RAA for SHZD32-1***

In order to test the specificity of RRC platform for SHZD32-1, all plant materials, including 3 samples of GM maize, 3 samples of GM cotton, 3 samples of GM rapeseed, 6 samples of GM soya, and non transgenic soya were extracted by the RDEM. Extracted DNA of these samples was amplified by RAA triggered by CHP for 20 min. The visual fluorescence result of RAA shown that bright green fluorescence was observed only in the tube containing SHZD32-1, while other tubes no visible fluorescence (Fig.S4A). Furthermore, all these samples were analyzed by conventional PCR and RT-PCR for specific detection of SHZD32-1. As expected, the detection results of conventional PCR and RT-PCR were consistent with those obtained using RRC platform, that only SHZD32-1 was be observed from these samples (Fig. S4A).

The haploid genome size of soybean was estimated to be 1115 Megabasepairs (Mbp), corresponding to a weight of 1.155 pg. The copy number of the haploid soybean genome was calculated by the weight of the soybean DNA divided by 1.155 pg.
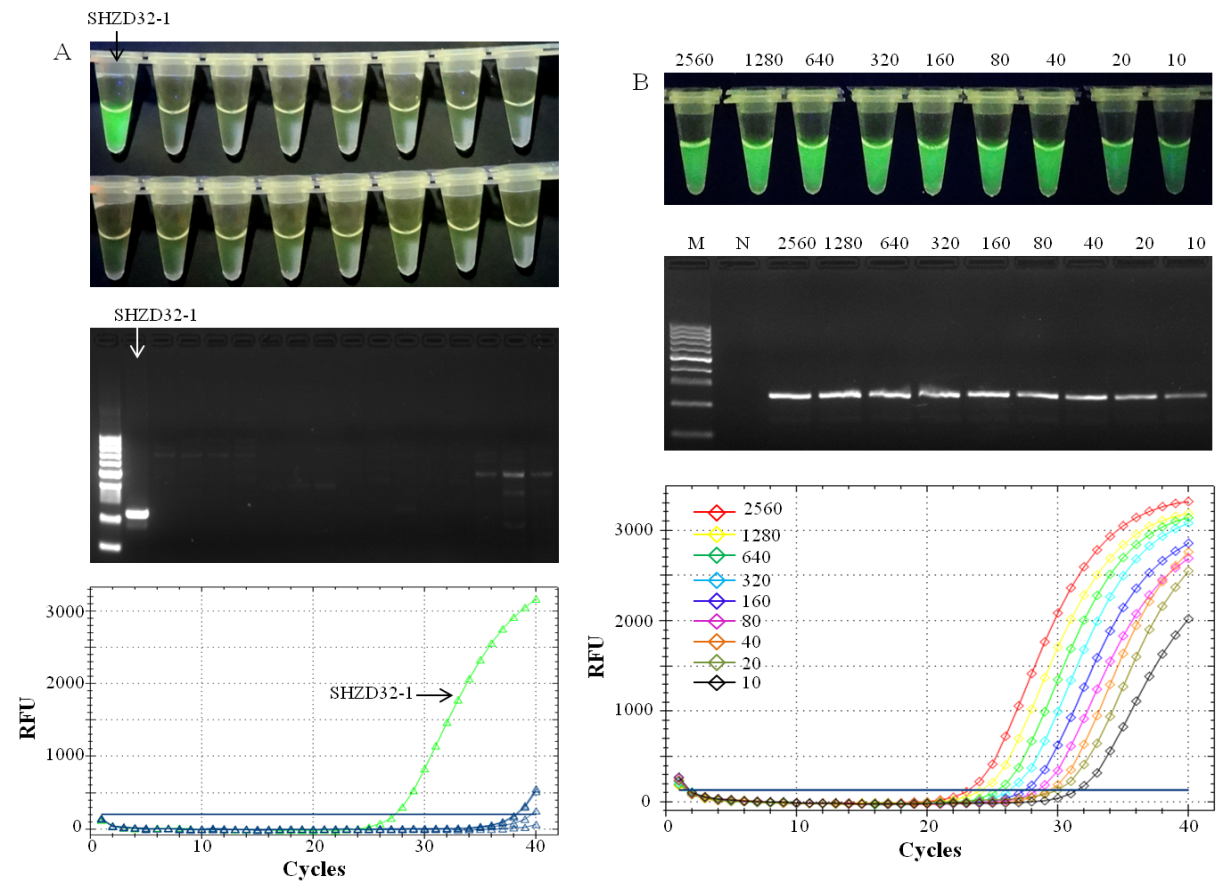
 The sensitivity of the RRC platform was tested with several dilutions of SHZD32-1 genomic DNA, at concentrations of 2560，1280, 640, 320, 160, 80, 40, 20, and 10 copies per reaction. The result of RAA visible fluorescence shown that bright green fluorescence was observed in all reaction, even at the low level of 10 copies/reaction (Fig. S4B). The sensitivity of the RRC platform was similar to the sensitivity of conventional PCR and RT-PCR (Fig. S4B).

Fig. S4. Specificity and sensitivity of the RRC platform. (A) Specificity of RRC platform (top panel), conventional PCR (middle panel), and RT-PCR (bottom panel). (B) Sensitivity of RRC platform (top panel), conventional PCR (middle panel), and RT-PCR (bottom panel). M: DL1000 DNA marker; N: No template control.

**References**

Wnag, X., Li, W., Xu, F., Shen, P., Yang, L, Chen, X., Xu, X., Peng, C., Liu, H., Ma, H., Qing, Y., 2017. Announcement by the Ministry of Agriculture No.1193-3-2009.

Wang, X., Chen, Y., Chen, X., Peng, C., Wang, L., Xu, X., Wu, J., Wei, W., Xu, J., 2020. A highly integrated system with rapid DNA extraction, recombinase polymerase amplification, and lateral flow biosensor for on-site detection of genetically modified crops. Analytica Chimica Acta 1109, 158-168.
